# Modulation of the Astrocyte-Neuron Lactate Shuttle System contributes to Neuroprotective action of Fibroblast Growth Factor 21

**DOI:** 10.7150/thno.44370

**Published:** 2020-07-09

**Authors:** Yan Sun, Yue Wang, Su-Ting Chen, Ying-Jie Chen, Jie Shen, Wen-Bing Yao, Xiang-Dong Gao, Song Chen

**Affiliations:** Jiangsu Key Laboratory of Druggability of Biopharmaceuticals, State Key Laboratory of Natural Medicines, School of Life Science and Technology, China Pharmaceutical University, Nanjing 211198, PR China.

**Keywords:** Alzheimer's disease, Astrocyte, FGF21, Neuroprotective, Neuron

## Abstract

A viewpoint considering Alzheimer's disease (AD) as “type 3 diabetes” emphasizes the pivotal role of dysfunctional brain energy metabolism in AD. The hormone fibroblast growth factor 21 (FGF21) is a crucial regulator in energy metabolism; however, our understanding of the therapeutic potential and mechanisms underlying the effect of FGF21 on neurodegeneration of AD is far from complete.

**Methods:** To further elucidate the effect of FGF21 on AD-related neurodegeneration, we used APP/PS1 transgenic mice to assess the effects of FGF21 on memory dysfunction, amyloid plaque pathology and pathological tau hyperphosphorylation. We also established an *in vitro* system to mimic astrocyte-neuron communication and an *in vivo* model of acute injury. Based on the *in vivo* and *in vitro* models, we analyzed the neuroprotective actions of FGF21 and pathways related to astrocyte-neuron communication and further focused on the astrocyte-neuron lactate shuttle system.

**Results:** Here, we report that FGF21 can ameliorate Alzheimer-like neurodegeneration in APP/PS1 transgenic mice. We detected defects in the astrocyte-neuron lactate shuttle system in the *in vivo* and *in vitro* models of AD and identified FGF21 as a neuroprotective molecule that can rescue these deficits. Administration of FGF21 can alleviate memory dysfunction, amyloid plaque pathology and pathological tau hyperphosphorylation, and the function of FGF21 in neurodegeneration is mediated in part by monocarboxylate transporters (MCTs). *In vivo* evidence also suggests that FGF21 acts centrally in mice to exert its effects on neurodegeneration and energy metabolism *via* its regulation of MCTs.

**Conclusions:** These results suggest that FGF21 alters metabolic parameters to mediate its neuroprotective functions. Modulation of the astrocyte-neuron lactate shuttle system can be one of the most efficient strategies for FGF21 in Alzheimer-like degeneration and contributes to improvements in brain metabolic defects and amyloid β-induced cytotoxicity. Our findings provide insights into the mechanisms underlying the effects of FGF21 on neurodegeneration and brain energy metabolism and suggest that FGF21 may have therapeutic value in the treatment of AD and other neurodegenerative diseases.

## Introduction

Currently, approved therapeutic options are still limited for treating neurodegenerative diseases, especially Alzheimer's disease (AD) 1. AD, the main cause of dementia, is one of the most common neurodegenerative diseases. The senile plaques formed by amyloid β-protein (Aβ) aggregation and neurofibrillary tangles composed of hyperphosphorylated tau are the two major hallmarks of AD pathologies 1-6. Unfortunately, in recent years, most mono-target drug candidates designed directly for Aβ or tau have not achieved desirable efficiency in clinical trials 7, 8. These results indicate that AD is far more complicated than we believe due to the multiple risk pathways involved. Among the potential risk factors contributing to AD, diabetes is now receiving increasing attention 9, 10. An initial Rotterdam study conducted over twenty years ago analyzed the relationship between diabetes mellitus and the risk of dementia, and this study first revealed the possibility that dementia can be considered one type of metabolic syndrome 11. Subsequently, numerous studies have supported this concept, and AD is even described as “type 3 diabetes” 12. This hypothesis prompted us to search for pharmacotherapy for AD from the pool of endogenous regulators of glucose metabolism, such as insulin, glucagon-like peptide-1, fibroblast growth factor 21 (FGF21), and so on 13-19.

FGF21 is an endocrine hormone that functions as a regulator in energy metabolism, and several target organs and tissues are involved in the diverse activities of FGF21 20-27. The ability to cross the blood-brain barrier (BBB) and the wide distribution of receptors allow FGF21 to exert direct effects in the central nervous system 28-32. Previously, we reported that peripheral administration of FGF21 ameliorates Aβ(25-35)-induced memory deficits in rats 16. However, the therapeutic potential and mechanisms underlying the action of FGF21 on neurodegeneration in AD urgently need to be further explored in a symptomatic transgenic animal model. According to the viewpoint considering AD as "type 3 diabetes", abnormal brain energy metabolism plays a pivotal role in the occurrence and development of AD 33, 34. The abnormal brain energy metabolism, especially the abnormal energy metabolism of neurons, leads to hippocampal neuron death and eventually AD 35-37. Furthermore, pathological Aβ and tau changes and neuronal energy metabolic dysfunction mutually reinforce each other to further aggravate AD. The brain energy metabolic network is complicated, and while neurons are affected by their own energy metabolic pathways, the energy interaction pathways between astrocytes and neurons are also crucial 36-41. Physically, the important role of astrocyte-neuron interactions in brain energy metabolism has been summarized and emphasized, and pathologically, extensive evidence indicates that disruption of astrocyte-neuron communications leads to neurodegenerative diseases 38, 42-44. In particular, there are many studies supporting the astrocyte-neuron lactate shuttle hypothesis 45-48. Astrocytes release high levels of lactate in the extracellular space, which can be utilized by neurons to meet energy requirements. The evidence from numerous studies supports lactate transfer from astrocytes to neurons and its importance for brain function, and the opposing view suggests that more evidence for the unidirectional flow of lactate from astrocytes to neurons is still needed 46, 47, 49, 50. Lactate has even been proven to be an effective neuroprotective agent, and exogenously administered lactate can diffuse through the astrocyte network and rescue neuronal activity during glucose deprivation 39. Previous study has shown that in the rat hippocampus, learning behavior increases the amount of lactate outside the astrocyte, and astrocyte-neuron lactate transport is the key for long-term memory formation 40. Our understanding of the mechanisms underlying the effects of FGF21 on AD and the astrocyte-neuron lactate shuttle system is far from complete, and whether FGF21 acts directly through the central nervous system in animal models of AD also needs to be further explored.

## Methods

### Transgenic mice

Six-month-old male APP/PS1 transgenic mice (B6C3-Tg (APPswe/PSEN1dE9)85Dbo/Nju) and age-matched wild-type (WT) littermates were obtained from the Model Animal Research Center of Nanjing University (Nanjing, China). All mice were housed in specific pathogen-free conditions at room temperature (22±1 °C) and a 12:12 h light-dark cycle at the Laboratory Animal Center of China Pharmaceutical University (Nanjing, China). The animal experiments were carried out in accordance with regulations of the Institutional Animal Care and Use Committee of China Pharmaceutical University.

For peripheral administration in transgenic mice, APP/PS1 mice were subcutaneously injected with FGF21 (5 mg/kg/d) or vehicle twice a day for one month, *n*=8-10.

For intracerebroventricular (ICV) injection of FGF21 into the transgenic mice *via* a minipump, FGF21 (0.4 μg/d over 14 days) or vehicle was given to the APP/PS1 mice. For the fibroblast growth factor receptor-1 (FGFR1) pAb group, the APP/PS1 mice were preinjected (ICV) with FGFR1 antibody (1 μg) (Abcam, Cambridge, USA) or vehicle. *n*=6-10.

### ICV administration of FGF21 *via* minipump

FGF21 (0.4 μg/d over 14 days) or vehicle was filled into a micro-osmotic pump (Alzet; Durect Co., Cupertino, USA) connected to the brain infusion cannula. The air bubble-free pump was incubated overnight in sterile saline at 37 °C according to the instruction manual. Mice were anesthetized before pump implantation. The brain infusion cannula was inserted stereotaxically into the right lateral ventricle (0.1 mm anteroposterior to bregma; 0.9 mm lateral from midline; 2.5 mm below the dura) by a brain stereotaxic instrument (RWD Life Science Co., Ltd., Shenzhen, China) before the pump was implanted under the skin of the back.

### *In vivo* blockade experiments

For the acute injury model induced by Aβ(25-35) and drug administration, siRNA was given (ICV) to C57BL/6J mice on day 1 and day 3, followed by ICV administration of Aβ(25-35) (10 nmol) and FGF21 (1 μg) on day 3. For the small molecule inhibitor group, the mice were preinjected (ICV) with PD173074 (25 μg) (Sigma-Aldrich, St. Louis, USA) prior to treatment with Aβ(25-35) and FGF21. The mice were sacrificed 4 days after the peptide injection for biochemical tests.

*In vivo* transfection experiments were performed by two ICV injections of 1 μL siRNA (Biomics Biotechnologies Co., Ltd., Nantong, China). Briefly, 40 μg siRNA was dissolved in 20 μL of endotoxin-free pure water and then mixed with Entranster^TM^ *in vivo* transfection reagent (Engreen Biosystem Co., Ltd., Beijing, China) at a ratio of 2:1. siRNA sequences: monocarboxylate transporter 2 (MCT2): sense 5'-CUGUCACAGUAUUCUUCAAdTdT-3', antisense 5'-UUGAAGAAUACUGUGACAGdTdT-3'; monocarboxylate transporter 4 (MCT4): sense 5'-GGAGCUUAUGCAUGAGUUUdTdT-3', antisense 5'-AAACUCAUGCAUAAGCUCCdTdT-3'; negative control: sense 5'-UUCUCCGAACGUGUCACGUdTdT-3', antisense 5'-ACGUGACACGUUCGGAGAAdTdT-3'.

### Cells and treatments

The astrocyte line (C6) and the neuronal line (PC12) were cultured in Dulbecco's modified Eagle's medium (DMEM, Gibco, Grand Island, USA) with 10% fetal bovine serum (FBS, Gibco) in 5% CO_2_ at 37 °C.

Primary hippocampal neurons were isolated and purified from day E18 embryos of Wistar rats. Cells were plated on poly-*D*-lysine (50 μg/mL)-coated 24-well plates containing DMEM supplemented with 10% FBS in 5% CO_2_ at 37 °C, and DMEM was replaced with Neurobasal-B27 medium (containing 0.5 mM L-glutamine) after 6 h. The medium was changed every two days, and neurons were used in co-culture studies after 8 days.

Primary astrocytes were isolated and purified from neonatal Wistar rats. Cells were plated on poly-*D*-lysine (50 μg/mL)-coated culture flasks in DMEM/F12 medium (1:1) supplemented with 10% FBS in 5% CO_2_ at 37 °C, and the medium was changed every two days. Astrocytes were purified by shaking at 220 rpm/min for 4 h on the 10th day of culture.

For astrocyte-conditioned medium (ACM) experiments (*n*=4), FGF21-ACM was transferred to neurons with Aβ(25-35) treatment.

For co-culture studies (*n*=3), neurons were plated in the bottom Transwell chamber (Corning Inc., Corning, USA) with Aβ(25-35) treatment, and astrocytes were seeded onto the top Transwell chamber with a 0.4 µm pore polyester membrane insert prior to FGF21 intervention.

For *in vitro* blockade experiments (*n*=3), the target and control siRNA (Biomics Biotechnologies Co., Ltd.) were transfected into astrocytes and neurons using Lipofectamine 3000 (Invitrogen, Carlsbad, USA) according to the manufacturer's guidelines. For the small molecule inhibitor group, MCT2 inhibitor AR-C155858 (1.25 nM; MedChemExpress, Monmouth Junction, USA) was used in the astrocyte-neuron co-culture system. siRNA sequences were as follows: MCT2: sense 5'-GGCUCAAGACAAGAUUCAAdTdT-3', antisense 5'-UUGAAUCUUGUCUUGAGCCdTdT-3'; MCT4: sense 5'-CGGUCUUUGUGGUGAGCUAdTdT-3', antisense 5'-UAGCUCACCACAAAGACCGdTdT-3'; negative control: sense 5'-UUCUCCGAACGUGUCACGUdTdT-3', antisense 5'-ACGUGACACGUUCGGAGAAdTdT-3'.

### Behavioural assessment

Learning and memory abilities were evaluated by the Morris water maze. The pool (diameter: 120 cm, height: 40 cm) (Beijing Zhongshidichuang Science and Technology Development Co., Ltd., Beijing, China) was filled with water at a constant temperature of 22 °C, and nontoxic white pigment was added to the water. The platform was placed in the center of the target quadrant 1 cm below the surface of the water. In the first five days of training, the animals were placed in the water, and the escape latency time was recorded when the animal found the underwater platform. During the training sessions, mice that failed to find the platform within 60 s were guided to and remained on the platform for 10 s. In the probe test on the sixth day, the platform was removed, and the number of crossing the original location of the platform and the time spent in the target quadrant within 60 s were recorded.

### Immunohistochemistry

Mouse brains were processed into 5 μm thick slices with paraffin embedding and dewaxed in a gradient concentration organic solvent. Tissue sections were placed in a microwave oven for antigen retrieval, immersed in 3% hydrogen peroxide solution at room temperature and protected from light for 25 min to block endogenous peroxidase. After the slices were dried, the primary antibody (anti-β-amyloid (6E10) (BioLegend, San Diego, USA), p-tau (Thr 181) (Cell Signaling Technology, Danvers, USA), MCT2 (ABclonal, Wuhan, China) and MCT4 (Santa Cruz Biotechnology, Santa Cruz, USA)) diluted in 5% bovine serum albumin (BSA) was added to cover the tissue. The sections were placed in a wet box and incubated overnight at 4 °C, and the HRP-conjugated secondary antibody and diaminobenzidine tetrahydrochloride were used. The nuclei were stained with hematoxylin, and the tissue sections were sealed with neutral gum. Images were captured using a compound microscope (COIC, Chongqing, China). The analysis of protein level was performed in ImageJ's “analyze particles” function used for counting relative areas, and thresholds were consistent across all groups.

### Immunofluorescence

Paraffin sections of brain tissue were dewaxed in different concentrations of organic reagents, and antigen retrieval was performed with ethylenediaminetetraacetic acid in a microwave oven. Tissue slices were incubated with an autofluorescence quencher for 5 min to eliminate autofluorescence. The primary antibody (anti-β-amyloid (6E10) (BioLegend), MCT4 (Santa Cruz Biotechnology) and GFAP (Abcam)) diluted in 5% BSA was added to cover the tissue after the slices were dried. Then, the slices were placed in the wet box and incubated overnight at 4 °C, and the immunoreaction was detected using the fluorescence-conjugated secondary antibody. The nuclei were stained with DAPI, and the tissue sections were sealed with an anti-fluorescence quencher. Images were captured using a confocal laser scanning microscope (Zeiss, Jena, Germany).

For thioflavin S (ThS) staining, after incubation with β-amyloid (6E10) antibody and the fluorescent secondary antibody, the tissue sections were stained for 8 min with a solution of 0.3% ThS in 50% ethanol. Images captured using a DeltaVision system (GE Healthcare, Little Chalfont, UK).

### Cell viability analysis

Cell viability was tested by 3-(4, 5-dimethylthiazol-2-yl)-2, 5-diphenyltetrazolium bromide (MTT, Sigma-Aldrich) assay. Briefly, 20 μL of 5 mg/mL MTT was added to the medium and incubated with cells for 4 h. Then, the medium containing MTT was removed, and dimethyl sulfoxide was added to each well. Absorbance values were detected at 570 nm and 630 nm (reference) by a microplate reader (Thermo Fisher Scientific, Waltham, USA).

### Cell apoptosis assay

Cells were double stained using an Annexin V-FITC/PI assay kit (Beyotime Institute of Biotechnology, Shanghai, China). Briefly, the cells were collected, treated with 1 μL of Annexin V-FITC staining solution and 2 μL of PI staining solution for 20 min at room temperature in the dark, and analyzed using flow cytometry (BD Biosciences, San Jose, USA).

### Western blot

Proteins from cell and tissue samples were extracted using RIPA buffer containing protease and phosphatase inhibitors (Merck Millipore, Billerica, USA). Proteins were separated by SDS-PAGE and then transferred to PVDF membranes (Merck Millipore). The membrane was blocked for 2 h at room temperature with 3% BSA and incubated with primary antibody overnight at 4 °C. After the membrane was washed with TBST, it was incubated with the secondary antibody for 1.5 h at room temperature. The immunological complexes were visualized by the ECL method using Tanon 5200 Multi Chemiluminescent Imaging System (Tanon, Shanghai, China). Analyses of protein levels were performed by using ImageJ.

The primary antibodies were as follows: anti-p-tau (Thr 181) (#12885S, Cell Signaling Technology), p-tau (Thr 205) (ab4841, Abcam), p-tau (Ser 404) (#20194S, Cell Signaling Technology), tau46 (#4019S, Cell Signaling Technology), β-actin (AC026, ABclonal), glucose transporter protein type 1 (GLUT1; ab115730, Abcam), lactate dehydrogenase A (LDHA; #2012S, Cell Signaling Technology), MCT4 (sc-376140, Santa Cruz Biotechnology), monocarboxylate transporter 1 (MCT1; AB3540P, Merck Millipore), glucose transporter protein type 3 (GLUT3; ab191071, Abcam), lactate dehydrogenase B (LDHB; ab85319, Abcam), MCT2 (A12386, ABclonal), FGFR1 (ab10646, Abcam), β-Klotho (KLB; SAB2108630, Sigma-Aldrich), p-AMPK (Thr172) (#2531, Cell Signaling Technology), AMPK (#2532, Cell Signaling Technology), p-mTOR (Ser2448) (#5536, Cell Signaling Technology), mTOR (#2972, Cell Signaling Technology), Beclin-1 (#3738, Cell Signaling Technology), and LC3B (#2775, Cell Signaling Technology).

### Quantitative real-time PCR (qRT-PCR)

Total RNA was extracted by an* EasyPure*^®^ RNA Kit (TransGen Biotech, Beijing, China) and reverse transcribed into cDNA using HiScript^®^ II Q RT SuperMix for qPCR (+gDNA wiper) (Vazyme Biotech Co., Ltd., Nanjing, China) according to the manufacturer's instructions. qRT-PCR was performed to detect the gene mRNA levels using ChamQ^TM^ Universal SYBR^®^ qPCR Master Mix (Vazyme Biotech Co., Ltd.). Primers were synthesized by Kingsray (Kingsray Biotechnology Co., Ltd., Nanjing, China). The qPCR conditions were as follows: 95 °C for 3 min and 40 cycles of 95 °C for 10 s and 60 °C for 30 s. Melting curves were tested to assess the accuracy of the PCR analysis. The 2^-ΔΔCt^ method was used to analyze the level of gene expression. The calculation methods for ΔCt and ΔΔCt values were as follows: ΔCt = Ct (target gene) - Ct (β-actin gene), ΔΔCt =ΔCt (treatment) -ΔCt (control). The primer sequences were as follows: excitatory amino acid transporter (EAAT) 1-Forward: ACCAAAAGCAACGGAGAAGAG, EAAT1-Reverse: GGCATTCCGAAACAGGTAACTC; EAAT2-Forward: GCACGAGAGCTATGGTGTATTAC, EAAT2-Reverse: GTTTGGGATTACCTGGGTGGA; EAAT3-Forward: CTTCCTACGGAATCACTGGCT, EAAT3-Reverse: CGATCAGCGGCAAAATGACC; alanine-serine-cysteine-1 transporter (SLC7A10/ASC-1)-Forward: GGGACTACGCCTATGTCACTG, SLC7A10-Reverse: ACCCACGTCAGGAGCATCA; neutral amino acid transporter (ASCT1)-Forward: GGCATCGCTGTTGCTTACTTC, ASCT1-Reverse: CGAGGAAAGAGTCCACTGTCT; phosphoglycerate dehydrogenase (PHGDH)-Forward: ATGGCCTTCGCAAATCTGC, PHGDH-Reverse: AGTTCAGCTATCAGCTCCTCC; MCT4-Forward: GACACGGCTTGGATCTCCTC, MCT4-Reverse: CATTCCCAGGGACGCAAAGAG; MCT2-Forward: GCTGGGTCGTAGTCTGTGC, MCT2-Reverse: ATCCAAGCGATCTGACTGGAG; 6-phosphofructo-2-kinase (PFKFB3)-Forward: CCCAGAGCCGGGTACAGAA, PFKFB3-Reverse: GGGGAGTTGGTCAGCTTCG.

### Lactate colorimetric assay

The astrocyte lysates or mediums from cells were added to 96-well plates and adjusted to 50 μL of reaction mix as described in the lactate colorimetric assay kit instructions (Biovision, Milpitas, USA). After incubation for 30 min at room temperature in the dark, the absorbance at 570 nm was measured using a microplate reader (Thermo Fisher Scientific).

### Measurement of adenosine triphosphate (ATP) levels

The cell/tissue sample was lysed, and 100 μL of working solution was added in accordance with the ATP assay kit instructions (Beyotime Institute of Biotechnology). Relative light unit values were detected using a Tecan Infinite 200 Pro microplate reader (Tecan, Männedorf, Switzerland).

### Measurement of reactive oxygen species (ROS) levels

The neurons were treated with the DCFH-DA probe (Beyotime Institute of Biotechnology) for 20 min at 37 °C and subsequently washed three times using serum-free medium. The ROS levels were analyzed by flow cytometry (BD Biosciences) or fluorescence microscopy (Olympus, Tokyo, Japan).

### Mitochondrial membrane potential detection

The neurons were incubated with 1 mL of JC-1 staining solution (Beyotime Institute of Biotechnology) at 37 °C for 20 min. Then, the supernatant was aspirated, and the cells were washed twice with JC-1 staining buffer. JC-1 is a fluorescent probe that detects changes in mitochondrial membrane potential, and the probe can be in two states. When the mitochondrial membrane potential increases, JC-1 aggregates can be formed, and red fluorescence can be generated. In contrast, when the mitochondrial membrane potential decreases, JC-1 tends to form monomers and produces green fluorescence. Mitochondrial membrane potential changes were analyzed using a confocal laser scanning microscope (Zeiss).

### Transmission electron microscopy

Autophagic vacuole formation was analyzed using transmission electron microscopy. Briefly, the tissue was embedded, ultrathin sectioned, stained and observed with a transmission electron microscope (JEOL, Tokyo, Japan).

### Statistical analysis

Data analysis was performed using GraphPad software. Data are represented as the mean ± standard error of the mean. Two-way analysis of variance followed by Tukey's test was used for the data from training sessions in the Morris water maze, and one-way analysis of variance followed by Tukey's test was used for other data. *p* values < 0.05 were considered significant.

## Results

### Peripheral administration of FGF21 alleviates AD lesions in APP/PS1 transgenic mice

**Figure 1:**
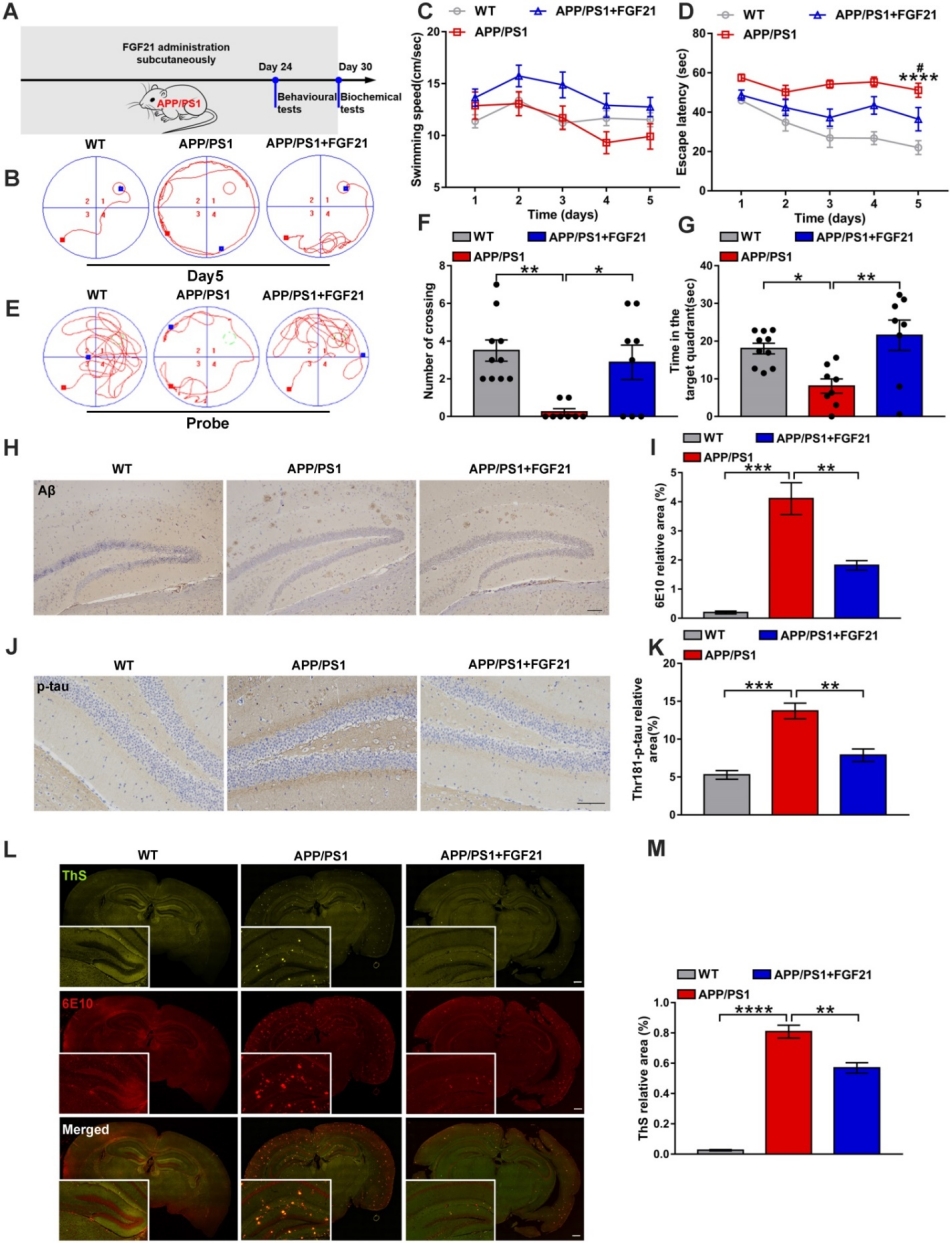
Peripheral administration of FGF21 attenuated memory impairment and AD-like pathologies in the APP/PS1 transgenic mice. **A.** Scheme for peripheral administration of FGF21 in the APP/PS1 mice. B-G. FGF21 (5 mg/kg/d, subcutaneously) was given to six-month-old male APP/PS1 transgenic mice for one month, and behavioral testing was performed. Representative swim paths on the fifth day of training (**B**) are shown, and swimming speed (**C**) and escape latency (**D**) during the first five days of training were analyzed. *n*=8-10. The *p*-value compared with the WT group is <0.0001 (****). The *p*-value compared with the APP/PS1+FGF21 group is <0.05 (^#^). Representative swim paths (**E**), number of crossing the original location of the platform (**F**) and time spent in the target quadrant (**G**) are shown for the probe trial. **H.** Aβ levels in the mouse brains were analyzed by immunostaining with 6E10, and representative images are shown (scale bar, 200 µm). **I.** Quantitative results for H. **J.** Thr-181-p-tau antibody was used to immunohistochemically detect tau hyperphosphorylation in mouse brains, and representative images are shown (scale bar, 100 µm). **K.** Quantitative analysis of phosphorylation levels of tau protein in J. **L.** Coimmunostaining with ThS and 6E10 for amyloid plaque in the mouse brains. Representative images are shown (scale bar, 1 mm). **M.** Quantitative analysis of amyloid plaque deposition in L. All data are presented as the mean ± SEM. * *p* < 0.05; ** *p* < 0.01; *** *p* < 0.001; **** *p* < 0.0001.

To confirm the therapeutic effects of peripheral administration of FGF21 in a symptomatic transgenic animal model of AD, we used six-month-old male APP/PS1 transgenic mice (subcutaneously injected with FGF21 at 5 mg/kg/d for one month) (Figure 1A). In the Morris water maze test, the APP/PS1 mice showed severe cognitive impairment compared with the WT mice. After 5 days of training, the learning abilities of the APP/PS1 mice injected with FGF21 were enhanced, and the escape latency was shortened compared to that of the APP/PS1 mice injected with vehicle only, while the swimming speeds showed no significant change (Figure 1B-D). In the final probe test (the platform was removed), the FGF21-treated APP/PS1 mice showed a significant improvement in the number of crossing the original location of the platform and the time spent in the target quadrant compared with vehicle-treated transgenic mice, suggesting that peripheral administration of FGF21 can alleviate the defect of the APP/PS1 mice in spatial memory learning (Figure 1E-G). To further study the mechanism underlying the effects of FGF21, we collected all the brain tissues of the mice for pathological examination (Figure 1H-M) after the behavioral test. We applied an antibody 6E10 to detect all forms of Aβ (including APP, oligomer and plaque) (Figure 1H, I, L, M) and ThS staining (Figure 1L-M) to quickly identify Aβ plaques in the brain. To confirm the effect of FGF21 on Aβ plaques, we also performed simultaneous colocalization of immunostaining and ThS staining (Figure 1L-M). We observed that FGF21 significantly decreased the brain Aβ burden in the APP/PS1 mice.

Studies have indicated that Aβ plaques promote tau protein pathology, neuronal damage, and eventually AD 51, 52. To explore the effect of FGF21 on tau phosphorylation, we used immunohistochemistry to analyze the extent of tau hyperphosphorylation in the brain by an antibody recognizing the phosphorylation of Thr-181. Tau lesions occurred in the APP/PS1 mice, and the tau phosphorylation-positive area was significantly reduced in the drug-administered group, indicating that FGF21 can retard the pathologic process related to tau in the APP/PS1 mice (Figure 1J-K). Due to its ability to cross the BBB, FGF21 may enter the brain tissue after subcutaneous injection and exert neuroprotective effects centrally in the APP/PS1 mice.

### Astrocytes can strengthen the protective effects of FGF21 on neurons

**Figure 2:**
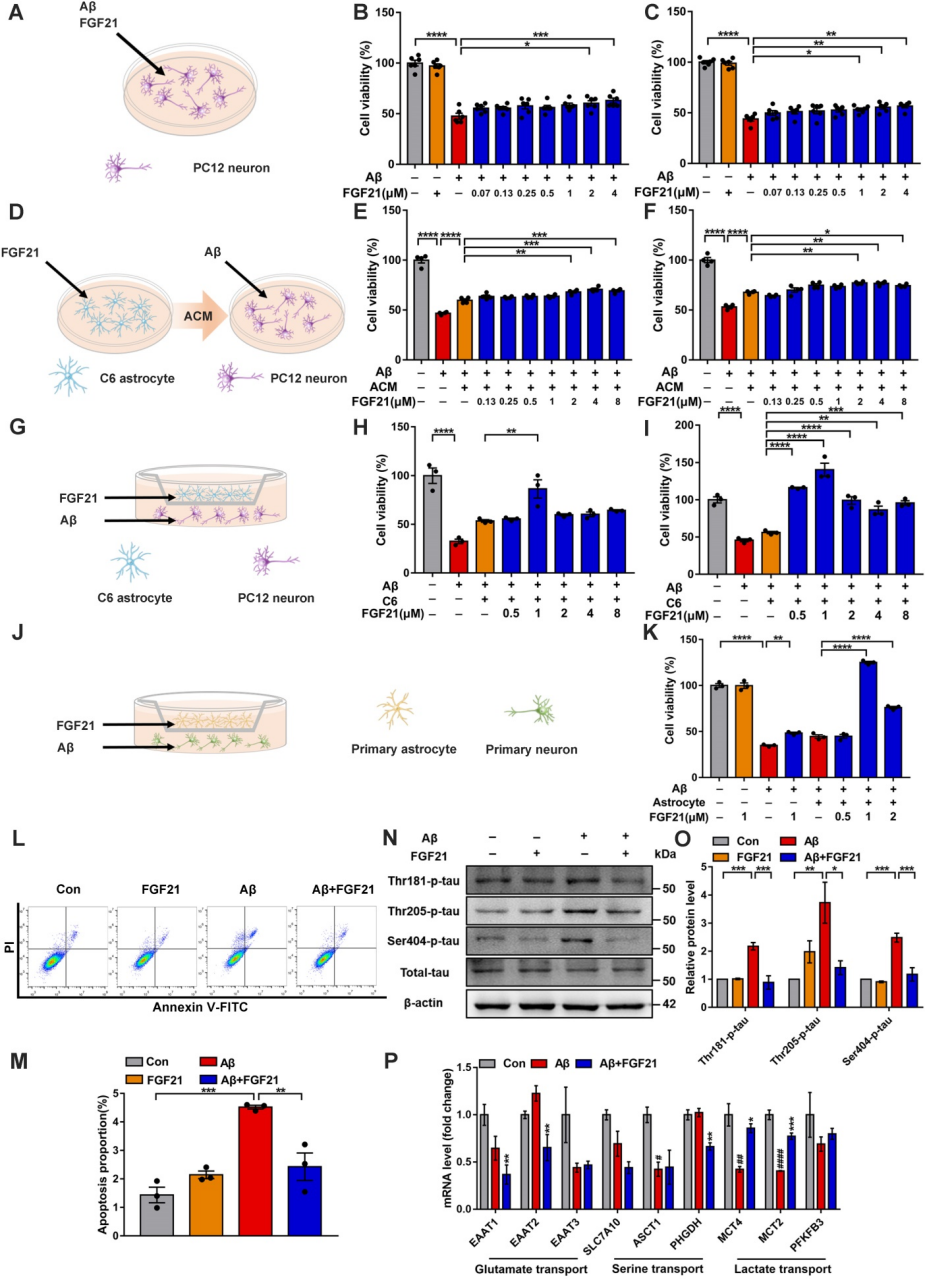
Astrocytes can strengthen the protective effects of FGF21 on neurons. **A.** Schematic diagram for treatments with FGF21 and Aβ(25-35) on PC12 neurons. **B-C.** Effects of different concentrations of FGF21 (0.07 µM, 0.13 µM, 0.25 µM, 0.5 µM, 1 µM, 2 µM, 4 µM) on Aβ(25-35) (0.0078 µM) induced PC12 neuronal injury at 24 h (B) and 48 h (C). *n*=6. **D.** Schematic diagram for treatments with FGF21-ACM and Aβ(25-35) on PC12 neurons. **E-F**. Effects of FGF21-ACM (concentrations of FGF21: 0.13 µM, 0.25 µM, 0.5 µM, 1 µM, 2 µM, 4 µM, 8 µM) on Aβ(25-35) (0.0078 µM) induced PC12 neuronal injury at 24 h (E) and 48 h (F). *n*=4. **G.** Schematic diagram for the establishment of the Transwell co-culture system with C6 astrocytes and PC12 neurons. **H-I.** Effects of different concentrations of FGF21 (0.5 µM, 1 µM, 2 µM, 4 µM, 8 µM) on Aβ(25-35) (0.0078 µM) induced PC12 neuronal injury at 24 h (H) and 48 h (I) in a Transwell co-culture system with C6 astrocytes and PC12 neurons. *n*=3. **J.** Schematic diagram for the establishment of the Transwell co-culture system with primary astrocytes and primary neurons. **K.** Effects of different concentrations of FGF21 (0.5 µM, 1 µM, 2 µM) on Aβ(25-35) (10 µM) induced primary neuron injury at 48 h in a Transwell co-culture system with primary astrocytes and primary neurons. *n*=3. **L.** PC12 cells in the co-culture system were double stained with Annexin V-FITC/PI for apoptotic detection, and representative images are shown. **M.** Quantitative results for L. *n*=3. **N.** The expression levels of Thr-181-p-tau, Thr-205-p-tau, Ser-404-p-tau and total tau protein in PC12 neurons in the Transwell co-culture system were detected by western blot, and representative images are shown. **O.** Quantitative results for N. *n*=3. Data are presented as the mean ± SEM. *p < 0.05; **p < 0.01; ***p < 0.001; ****p < 0.0001. **P**. C57 mice were injected with Aβ(25-35) (10 nmol) and FGF21 (1 µg) in the lateral ventricle. After 4 days, the relative mRNA levels of EAAT1, EAAT2, EAAT3, SLC7A10, ASCT1, PHGDH, MCT4, MCT2 and PFKFB3 in the hippocampus were determined by qRT-PCR. *n*=3. Data are presented as the mean ± SEM. ^#^*p* < 0.05, ^##^*p* < 0.01, ^####^*p* < 0.0001, compared to the Control group; *p < 0.05; **p < 0.01; ***p < 0.001, compared to the Aβ group.

To further confirm the direct action of FGF21 on brain cells, we conducted studies based on cell models. FGFR1 mainly mediates FGF21 signaling, and the coreceptor KLB is also required for the initiation event 29, 53. Thus, we first tested the existence of FGFR1 and KLB (Figure S1A-C). The evidence indicated that FGFR1 and KLB were expressed in cell lines (Figure S1A), primary neurons and astrocytes (Figure S1B) and brain tissues (Figure S1C). In this study, the FGF21-treated PC12 neurons showed increased cell viability against Aβ(25-35) toxicity (Figure 2A-C). Interestingly, for the Aβ(25-35)-induced model cells, ACM stimulated with FGF21 (FGF21-ACM) further increased the neuronal viability (Figure 2D-F) compared with FGF21-alone treatment (Figure 2A-C). Astrocytes may play a crucial role in the protective action of FGF21 on neurons. In the present study, ACM collected after treatment with FGF21 for 72 h significantly ameliorated the PC12 neuronal injury induced by Aβ(25-35); furthermore, ACM alone could also exert protective effects to a certain extent, but the effects were weaker (Figure 2E-F). Therefore, we further presumed that FGF21 may stimulate the secretion of astrocyte-derived factor, which can be beneficial for neurons. To mimic the *in vivo* situation as closely as possible, in this study, we established an *in vitro* system using Transwells (Figure 2G). In the Transwell system, 1 μM FGF21 significantly rescued the neuronal injury induced by Aβ(25-35) (Figure 2G-K). We confirmed this function of FGF21 in both the cell lines (Figure 2G-I) and primary cells (Figure 2J-K) for neurons and astrocytes in the Transwell system. In the Transwell co-culture system, to investigate the effect of FGF21 on Aβ(25-35)-induced apoptosis, we stained PC12 neurons using Annexin V-FITC/PI and analyzed them by flow cytometry (Figure 2L-M). The results showed that the proportion of apoptotic cells induced by Aβ(25-35) can be ameliorated by FGF21. Tau pathology, oxidative stress and mitochondrial damage are considered the main events along with Aβ insults in the progression and development of AD 3, 4, 54. To further analyze the variations in tau pathologies in the Transwell co-culture system, we collected protein samples from the PC12 neurons, and changes in tau phosphorylation were detected (Figure 2N-O). Our results showed that in the Transwell *in vitro* model, FGF21 could reduce the tau hyperphosphorylation caused by Aβ(25-35) at the Thr 181, Thr 205, and Ser 404 sites. Using fluorescent probes, we also assessed the ROS levels and change in the mitochondrial membrane potential in PC12 cells of the co-culture system (Figure S2A-D). The results showed that Aβ(25-35) aggravated excessive ROS production in neurons, while FGF21 significantly reduced the ROS levels, indicating that FGF21 could attenuate the oxidative damage caused by Aβ(25-35) (Figure S2A-C). Detection of mitochondrial membrane potential changes by the JC-1 fluorescent probe showed that FGF21 increased the Aβ(25-35)-induced downregulation of mitochondrial membrane potential in PC12 cells (Figure S2D). Thus, in the Transwell co-culture system, FGF21 could rescue neurons and attenuate the cell apoptosis, tau pathology, oxidative stress and mitochondrial damage induced by Aβ(25-35). The current data identify a critical role of astrocytes in the neuroprotective function of FGF21. FGF21 treatment alone did not significantly affect C6 cell viability (Figure S3A), so we presumed that FGF21 may promote the secretion of astrocytes, and this key factor played an important role in astrocyte-neuron communication and strengthened the effect of FGF21 on neurons. To further explore the mechanism underlying astrocyte-mediated FGF21 protection of neurons, we examined the alterations in the mRNA levels of multiple pathways related to astrocyte-neuron communication. We analyzed the effects of FGF21 on glutamate transport (EAAT1, EAAT2 and EAAT3), serine transport (SLC7A10/ASC-1, ASCT1 and PHGDH) and lactate transport (MCT4, MCT2 and PFKFB3) in the hippocampus with injury induced by Aβ(25-35), and the results suggested that astrocyte-neuron lactate transport may be crucial for the neuroprotective action of FGF21 (Figure 2P).

### Astrocyte-neuron lactate shuttle system defects in models of AD can be rescued by FGF21

**Figure 3:**
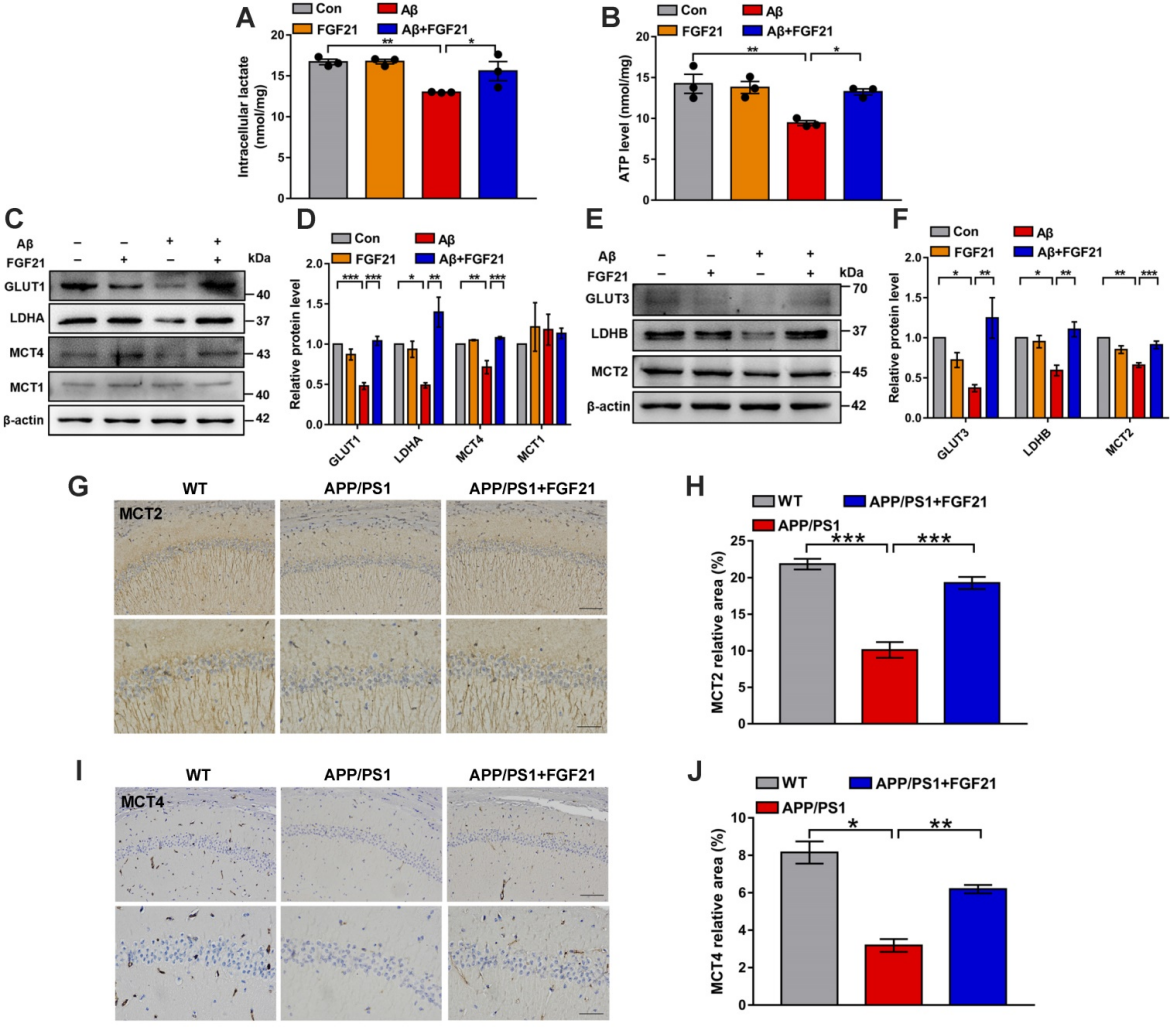
FGF21 rescued abnormal astrocyte-neuron lactate shuttling in the co-culture system and the APP/PS1 transgenic mice. **A-B.** In the co-culture system, the cells were treated with Aβ(25-35) and/or FGF21, and after 48 h, the intracellular lactate levels in C6 cells (A) and ATP levels in PC12 cells (B) were detected. *n*=3. **C.** The expression levels of GLUT1, LDHA, MCT4 and MCT1 in C6 cells in the co-culture system were detected by western blot, and representative images are shown. **D.** Quantitative results for C. *n*=3. **E.** The expression levels of GLUT3, LDHB and MCT2 in PC12 cells in the co-culture system were detected by western blot, and representative images are shown. **F.** Quantitative results for E. *n*=3. **G.** MCT2 levels in the mouse brains were analyzed by immunostaining, and representative images are shown (scale bar, upper images: 100 µm; lower images: 50 µm). **H.** Quantitative results for G. **I.** MCT4 levels in the mouse brains were analyzed by immunostaining, and representative images are shown (scale bar, upper images: 100 µm; lower images: 50 µm). **J.** Quantitative results for I. All data are presented as the mean ± SEM. *p < 0.05; **p < 0.01; ***p < 0.001.

The data indicated the vital importance of lactate shuttling between astrocytes and neurons for the effects of FGF21 on neurons. Between astrocytes and neurons, lactate shuttling contributed to their energy communication. Given that FGF21 is known as a key regulator of energy metabolism, we focused on astrocyte-neuron metabolic cooperation. Previous studies showed the high energy requirement in neurons in the brain and energy metabolic abnormalities in the process of cognitive impairment 34, 36-40. To understand how the neuron meets its energy demands, the astrocyte-neuron lactate shuttle model proposed by Pellerin and Magistretti has been widely accepted 45. However, determination of the exact role of the astrocyte-neuron lactate shuttle system in neurodegeneration requires more in-depth and systemic studies. Here, in the Transwell co-culture system in the present study, we first detected the levels of intracellular lactate in C6 astrocytes and the levels of intracellular ATP in PC12 neurons (Figure 3A-B). Medium from C6 cells treated with Aβ(25-35) and/or FGF21 has also been analyzed for lactate level (Figure S3B). We found that Aβ(25-35) caused a significant decrease in the levels of lactate and ATP, while FGF21 alleviated the abnormalities of the levels of these metabolic substances, suggesting that FGF21 can provide energy to neuronal cells by strengthening the supply of the energy substrate lactate from astrocytes to neurons. To further confirm the potential molecular targets in the astrocyte-neuron lactate shuttle system involved in the *in vitro* model and the action of FGF21, we then examined the expression levels of GLUT1, LDHA, MCT4 and MCT1 (Figure 3C-D). The results showed that FGF21 returned the expression levels of GLUT1, LDHA and MCT4 disrupted by Aβ(25-35) to normal levels and that MCT1 did not seem to participate in this process. FGF21 may promote glycolysis in C6 cells and accelerate the production of lactate while promoting the transfer of lactate from C6 cells. We also found that FGF21 can rescue the deficits in the pathways for lactate transfer into PC12 cells and the utilization of lactate in these cells by analyzing the expression levels of MCT2 and LDHB (Figure 3E-F). Monocarboxylate transporters (MCTs) play key roles in connecting the crosstalk between astrocytes and neurons *via* lactate; thus, we further examined the effects of FGF21 on MCTs *in vivo* using transgenic mice. MCTs were detected by immunostaining in the brain of the APP/PS1 mice (Figure 3G-J and Figure S4A). The results showed that MCT4 and MCT2 expression deficits were also detected in mouse models of AD and that FGF21 can alleviate the abnormal expression of MCT4 and MCT2. Taken together, these *in vitro* and* in vivo* results confirmed astrocyte-neuron lactate shuttle system defects in the AD models and suggested that the defects can be rescued by FGF21.

### *In vivo* evidence for FGF21 acting centrally to ameliorate the AD-related degeneration in transgenic mice

**Figure 4:**
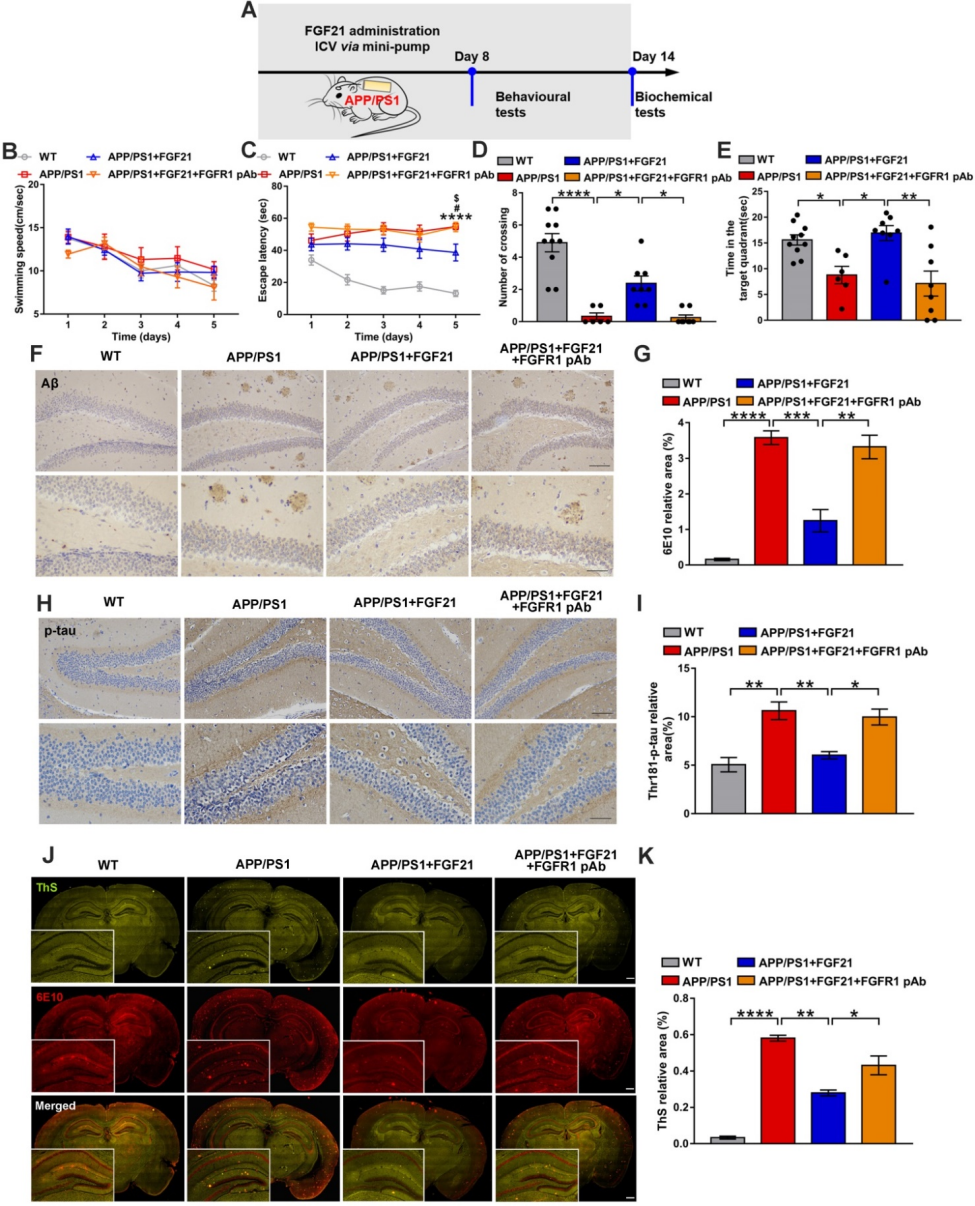
Central administration of FGF21 attenuated memory impairment and AD pathologies in the APP/PS1 transgenic mice. **A.** Scheme for ICV administration of FGF21 in the APP/PS1 mice. **B-E.** Six-month-old male APP/PS1 transgenic mice were administered FGF21 (ICV, 0.4 µg/d) for 14 days by using a micro-osmotic pump, and in the FGFR1 pAb group, the antibody was injected 10 min before FGF21 administration. The Morris water maze test was performed 7 days after surgery. Swimming speed (B) and escape latency (C) during the first five days of training were analyzed. *n*=6-10. The APP/PS1 *vs* WT* p*-value is <0.0001 (****). The APP/PS1 *vs* APP/PS1+FGF21 *p*-value is <0.05 (^#^). The APP/PS1+FGF21+FGFR1 pAb *vs* APP/PS1+FGF21 *p*-value is <0.05 (^$^). The number of crossing the original location of the platform (D) and time spent in the target quadrant (E) during the probe trial are shown. **F.** Aβ levels in the mouse brains were analyzed by immunostaining with 6E10, and representative images are shown (scale bar, upper images: 100 µm; lower images: 50 µm). **G.** Quantitative results for F. **H.** Thr-181-p-tau antibody was used to immunohistochemically detect tau hyperphosphorylation in mouse brains, and representative images are shown (scale bar, upper images: 100 µm; lower images: 50 µm). **I.** Quantitative analysis of phosphorylation levels of tau protein in H. **J.** Coimmunostaining with ThS and 6E10 for amyloid plaque in the mouse brains. Representative images are shown (scale bar, 1 mm). **K.** Quantitative analysis of amyloid plaque deposition in J. All data are presented as the mean ± SEM. * *p* < 0.05; ** *p* < 0.01; *** *p* < 0.001; **** *p* < 0.0001.

The *in vivo* data in the present study already showed that FGF21 can rescue neurodegeneration in the mouse model of AD by rescuing astrocyte-neuron lactate shuttle system defects in the brain. Although FGF21 can cross the BBB and it was presumed that FGF21 may act centrally, more direct* in vivo* evidence is still needed. To investigate the direct effects of FGF21 in the central nervous system, we administered FGF21 (0.4 μg/d) for 14 days in the lateral ventricles of the APP/PS1 mice by using a micro-osmotic pump and infusion cannula (Figure 4A). The Morris water maze test was initiated 7 days after surgery (Figure 4B-E). The results showed that ICV injection of FGF21 alleviated learning deficits in the APP/PS1 mice. A FGFR1 pAb was injected 10 min before FGF21 administration, and the FGFR1 pAb-treated APP/PS1 mice failed to learn the task after 5 days of training (Figure 4C). In the final probe test, FGF21 administration increased the residence time in the target quadrant and the number of crossing the original location of the platform in the APP/PS1 model, suggesting that FGF21 could alleviate cognitive impairment by directly acting on the central nervous system (Figure 4D-E). These effects of FGF21 were abolished by ICV pretreatment with FGFR1 pAb, which blocked the FGF21/FGFR1 signaling pathway. We also found that ICV injection of FGF21 decreased the Aβ plaque burden and hyperphosphorylation of tau (Figure 4F-K). The data demonstrated that FGF21 could exert anti-AD effects directly in the brain and that FGFR1 in the central nervous system was critical for the effect of FGF21 on AD.

### MCTs play key roles in FGF21, exerting beneficial effects on neurodegeneration and brain energy metabolism

**Figure 5:**
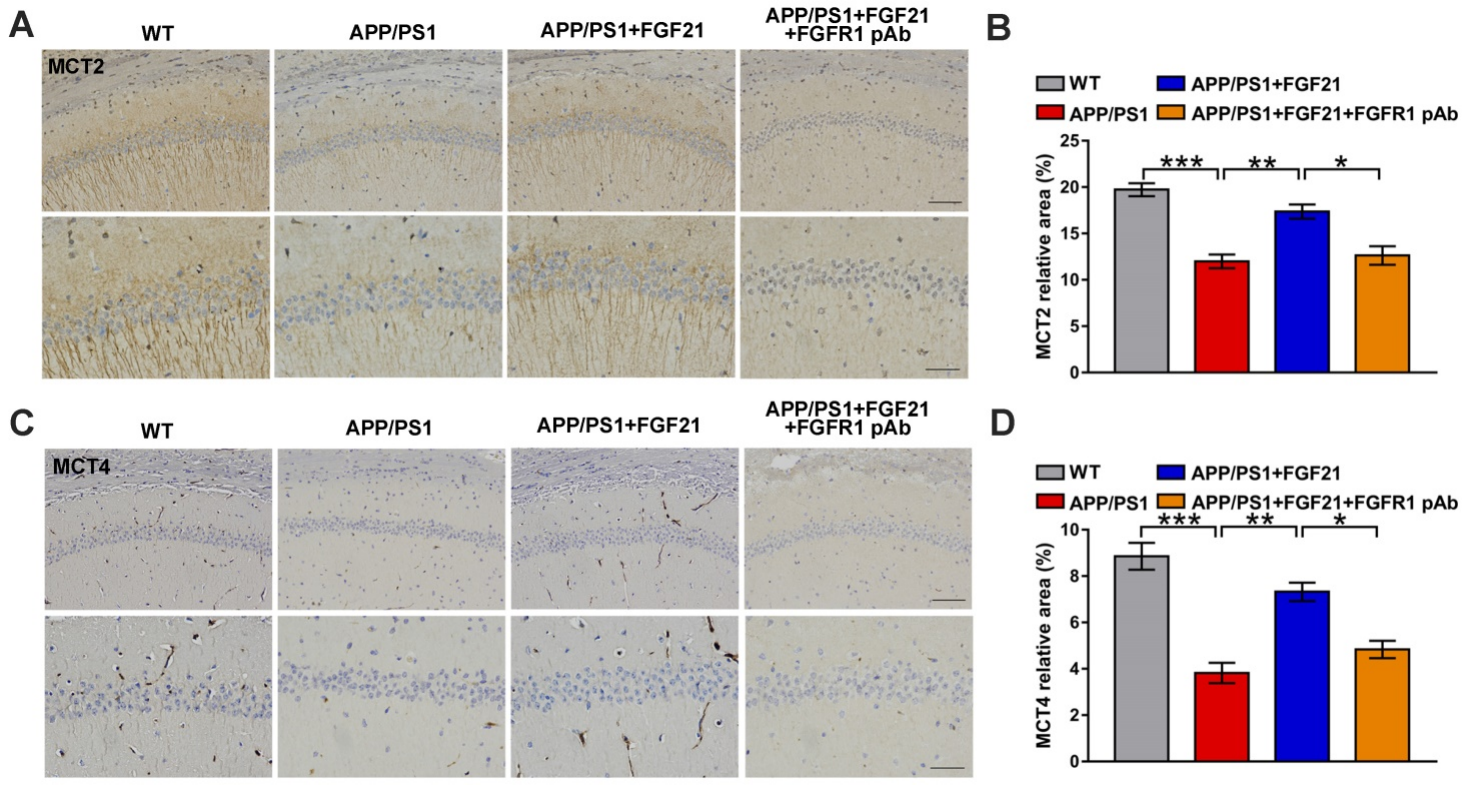
Central administration of FGF21 relieved MCT abnormalities* in vivo*. **A.** MCT2 levels in the mouse brains were analyzed by immunostaining, and representative images are shown (scale bar, upper images: 100 µm; lower images: 50 µm). **B.** Quantitative results for A. **C.** MCT4 levels in the mouse brains were analyzed by immunostaining, and representative images are shown (scale bar, upper images: 100 µm; lower images: 50 µm). **D.** Quantitative results for C. All data are presented as the mean ± SEM. * *p* < 0.05; ** *p* < 0.01; *** *p* < 0.001.

The current data indicated that MCTs in the astrocyte-neuron lactate shuttle may mediate the protective effects of FGF21 in the AD model. Both peripheral (Figure 3G-J and Figure S4A) and central administration (Figure 5A-D and Figure S4B) of FGF21 could regulate MCT expression in the brains of the APP/PS1 mice, and blocking FGFR1 could abolish the regulatory effects of FGF21 on abnormal levels of MCTs (Figure 5A-D and Figure S4B).

**Figure 6:**
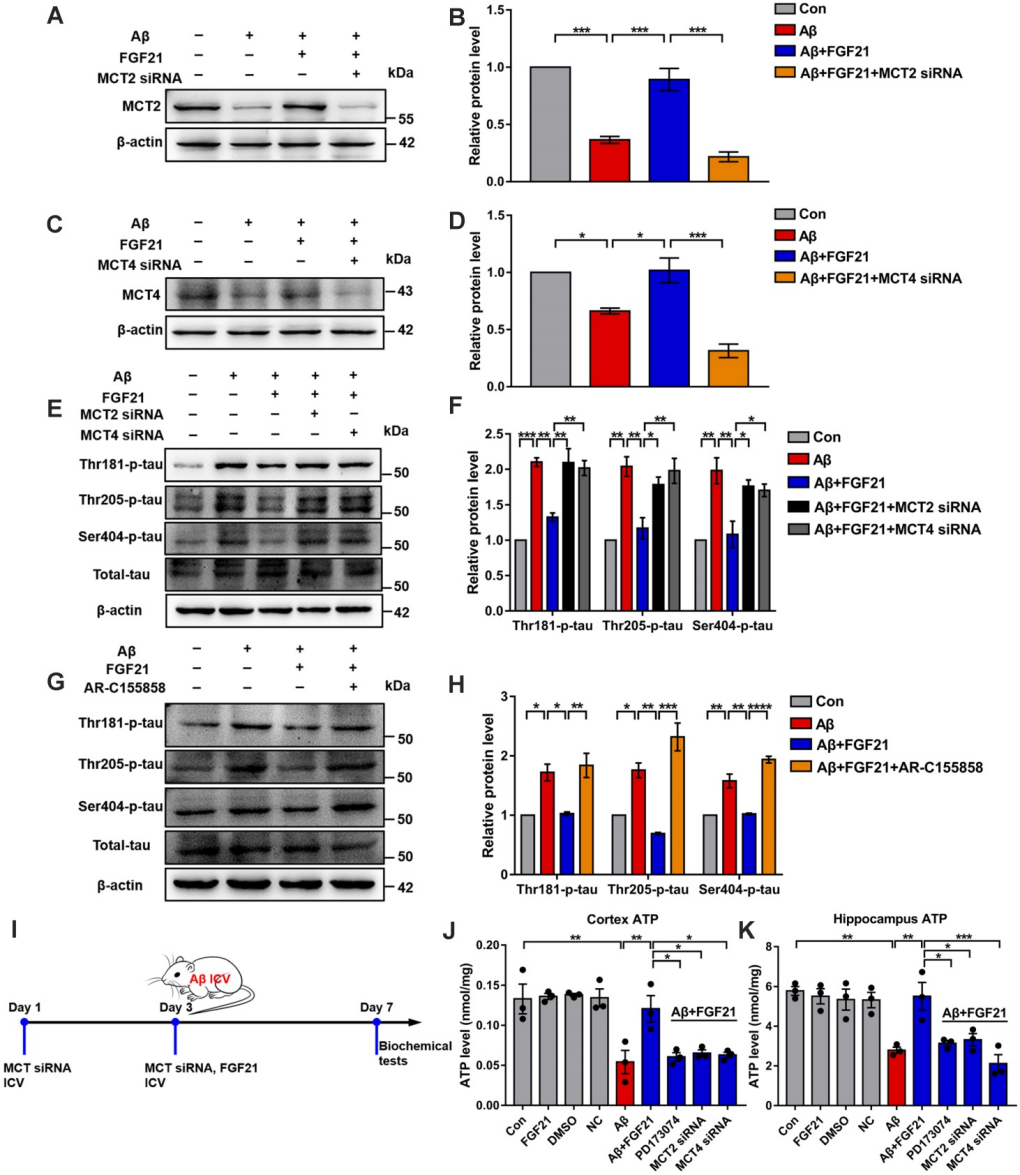
The roles of MCTs in the beneficial effects of FGF21. **A.** MCT2 expression of PC12 cells in a co-culture *in vitro* model was analyzed by western blot following transfection with MCT2 siRNA, and representative images are shown. **B.** Quantitative results for A. *n*=3. **C.** MCT4 expression in C6 cells in a co-culture *in vitro* model was analyzed by western blot following transfection with MCT4 siRNA, and representative images are shown. **D.** Quantitative results for C. *n*=3. **E.** Thr-181-p-tau, Thr-205-p-tau, Ser-404-p-tau and total tau levels of PC12 cells in a co-culture *in vitro* model were analyzed by western blot following transfection with MCT2 siRNA and MCT4 siRNA, respectively, and representative images are shown. **F.** Quantitative results for E. *n*=3. **G.** Thr-181-p-tau, Thr-205-p-tau, Ser-404-p-tau and total tau levels of PC12 cells in a co-culture *in vitro* model were analyzed by western blot following administration of the MCT2 inhibitor (AR-C155858, 1.25 nM), and representative images are shown. **H.** Quantitative results for G. *n*=3. **I.** Scheme for MCT siRNA* in vivo* transfection and FGF21 administration in mice. **J-K.** MCT siRNA was injected into the lateral ventricle of mice twice (on day 1 and day 3), followed by ICV administration of Aβ(25-35) (10 nmol) and FGF21 (1 μg) on day 3. For the FGFR1 inhibitor group, the inhibitor PD173074 (25 μg) was administered 10 min earlier than Aβ(25-35) and FGF21 injections (on day 3). On day 7, ATP levels in the cortex (J) and hippocampus (K) were detected. *n*=3. All data are presented as the mean ± SEM. * *p* < 0.05; ** *p* < 0.01; *** *p* < 0.001; **** *p* < 0.0001.

To further explore the mechanisms underlying the effects of FGF21 in AD by regulating the astrocyte-neuron lactate shuttle system, we blocked MCTs *in vitro* and* in vivo* by using siRNA (Figure S5). MCT4 and MCT2 were silenced in the Transwell co-culture system by transfecting MCT4 siRNA in C6 cells and transfecting MCT2 siRNA in PC12 cells, respectively (Figure 6A-D). siRNA silencing abolished the effects of FGF21 on tau protein hyperphosphorylation in the *in vitro* model (Figure 6E-F). We also blocked MCTs by using the MCT2 inhibitor AR-C155858 in the Transwell co-culture system. Compared to the group treated with FGF21 only, in the inhibitor and FGF21 cotreated group, the phosphorylation levels of tau protein (Thr 181, Thr 205 and Ser 404) were increased, and the protective effects of FGF21 on tau pathology disappeared (Figure 6G-H). In the *in vivo* system, we injected MCT siRNA into the lateral ventricle to analyze its influence on the efficacy of FGF21 therapy for ATP abnormalities in the mouse brain with injury induced by Aβ(25-35) (Figure 6I). The results showed that FGF21 therapy attenuated the ATP abnormalities in the injured cortex and hippocampus, while similar to the FGFR1 inhibitor, MCT siRNA also partly abolished the effects of FGF21 (Figure 6J-K).

## Discussion

Our understanding of AD is evolving from a “brain-only disease” to a “metabolic-cognitive syndrome” 9-12, 55, 56. AD is even referred to as “type 3 diabetes”, and a growing body of evidence suggests that abnormal brain energy metabolism is one of the main risk factors in the development of AD 12, 33.

Potential abnormalities in brain energy balance and metabolic disorders are commonly found in the AD brain. FGF21 is secreted from the liver, especially in response to glucose metabolic disorders 57. Overlaps in FGF21 expression and function as well as discrepancies exist between mice and humans; nevertheless, there is a multifunctional liver-brain axis with FGF21 as a pivotal player in mice and humans 58. Interestingly, a recent study reported that FGF21 can also be produced by neurons in response to mitochondrial dysfunction 59. Katsu-Jiménez *et al*. found that FGF21 can enhance the ability of cortical neurons to utilize ketone body through activation of AMP-dependent kinase 60. FGF21 directly acts in the brain to increase the insulin sensitivity and metabolic rate in rats with diet-induced obesity 30. Douris *et al*. confirmed that FGF21 can act in the brain to activate the sympathetic nervous system and induce adipose tissue thermogenesis 61. FGF21, a hormone that can act on the nervous system through interacting with the receptor, acted centrally to exert its effects on sympathetic nerve activity, energy expenditure and body weight 20. FGF21 can protect aging mouse brains against D-galactose-induced injury by decreasing the formation of advanced glycation end products, improving behavioral performance and attenuating oxidative stress-induced damage 18.

Studies have also indicated that FGF21 can protect the BBB through the FGF21/FGFR1/KLB signal axis, and alleviate neurological injury and prevent cognitive decline by repairing brain mitochondrial damage, altering hippocampal synaptic plasticity and ameliorating cell apoptosis 17, 19, 53, 62. Recently, we reported that peripheral administration of FGF21 ameliorates Aβ(25-35)-induced memory deficits in rats 16. However, the mechanisms underlying the effect of FGF21 on AD-like degeneration in the brain need to be further explored. We report here that FGF21 can act centrally against AD lesions in transgenic mice, and intervention with FGF21 shows promise for the amelioration of neurodegeneration. We demonstrated that FGF21 can exert anti-AD effects through peripheral administration in the APP/PS1 mice. Given its ability to cross the BBB 28, FGF21 may enter the brain after subcutaneous injection and centrally exert neuroprotective effects. However, more direct *in vivo* evidence is still needed, and in this study, we further demonstrated that FGF21 can also exert anti-AD effects through central administration in the APP/PS1 mice. The data indicated that FGF21 could exert anti-AD effects directly in the brain and that FGFR1 in the central nervous system was critical for the activity of FGF21 in AD. Aerobic glycolysis, lactate production and lactate transport between astrocytes and neurons in the brain are strongly associated with memory, and several studies have implicated alterations in lactate metabolism in AD 40, 63-65. Liguori *et al*. found that in AD, higher levels of cerebrospinal fluid AD biomarkers, including total tau and phosphorylated-tau, corresponded to lower concentrations of lactate, suggesting links between the pathological processes present in AD and impairment of neuronal energy metabolism 64. MCT4 and MCT2 are involved in the transfer of lactate from astrocytes to neurons in the brain, and MCTs in the central nervous system may show impaired expression in AD 66-69; thus, modulating their expression levels represents a potential strategy for neuroprotection 70, 71. In the context of AD, several studies have implicated changes in MCT expression as possible etiological factors 66-69. Lu *et al*. reported that downregulated MCT2 expression in the cerebral cortex and hippocampus in an Aβ(25-35)-treated rat model of AD may be correlated with the pathologic progression of AD, and the MCT2 protein level in the APP/PS1 transgenic mice was lower than that in the WT C57BL/6J mice 66, 67. Nitric oxide can contribute to the development of AD *via* downregulation of MCT1, and MCT1 may be a potential target for the treatment of AD 68. It was previously shown that enhancing the expression of MCTs can be a good neuroprotective strategy 70, 71. For example, overexpression of MCT2 in neurons can exert neuroprotective effects against an excitotoxic insult 70, and the recombinant *Lonomia obliqua* Stuart-factor activator showed neuroprotective effects on supplement-deprived mouse cultured cortical neurons *via* maintenance of the MCT2 protein levels 71. Our data support the key role of the astrocyte-neuron lactate shuttle in responding to metabolic abnormalities in neurodegeneration of AD and the effect of FGF21 in the central nervous system. Several previous studies have indicated the close ties between autophagy and FGF21 activity 72-75. In the *in vitro* and* in vivo* models in the present study, we also found that the classic autophagic response was strengthened *via* FGF21 administration (Figure S6). Thus, we speculate that FGF21 corrects various metabolic parameters to mediate its neuroprotective functions by inducing activation of multiple pathways. The modulation of the astrocyte-neuron lactate shuttle system may be one of the most efficient strategies for FGF21 in the initial stage of Alzheimer-like degeneration, and mutual promotions between this system and other classical pathways, such as autophagy induced by FGF21, may also contribute to the improvements in brain metabolic defects and Aβ-induced cytotoxicity.

## Conclusions

In summary, our findings suggest that FGF21 induces metabolic parameter corrections to mediate its neuroprotective functions. Modulation of the astrocyte-neuron lactate shuttle system can be one of the most efficient strategies for FGF21 in Alzheimer-like degeneration and contributes to improvements in brain metabolic defects and amyloid β-induced cytotoxicity. Our findings provide insights into the mechanisms underlying the actions of FGF21 on neurodegeneration and brain energy metabolism, and suggest that FGF21 may have potential therapeutic value in the treatment of AD and other neurodegenerative diseases.

## Supplementary Material

Supplementary figures.

## Abbreviations

AD: Alzheimer's disease
FGF21: fibroblast growth factor 21
MCTs: monocarboxylate transporters
Aβ: amyloid β-protein
BBB: blood-brain barrier
WT: wild-type
ICV: Intracerebroventricular
FGFR1: fibroblast growth factor receptor-1
DMEM: Dulbecco's modified Eagle's medium
FBS: fetal bovine serum
ACM: astrocyte-conditioned medium
BSA: bovine serum albumin
ThS: thioflavin S
MTT: 3-(4, 5-dimethylthiazol-2-yl)-2, 5-diphenyltetrazolium bromide
KLB: β-Klotho
LDHA: lactate dehydrogenase A
LDHB: lactate dehydrogenase B
GLUT1: glucose transporter protein type 1
GLUT3: glucose transporter protein type 3
EAAT: excitatory amino acid transporter
SLC7A10/ASC-1: alanine-serine-cysteine-1 transporter
ASCT1: neutral amino acid transporter
PHGDH: phosphoglycerate dehydrogenase
PFKFB3: 6-phosphofructo-2-kinase
ATP: adenosine triphosphate
ROS: reactive oxygen species
