# Supplementary material for: Modulation of the Astrocyte-Neuron Lactate Shuttle System contributes to Neuroprotective action of Fibroblast Growth Factor 21
Source: Theranostics. 2020 Jul 9;10(18):8430–45. doi: 10.7150/thno.44370 (PMC7381735; doi:10.7150/thno.44370)
Supplement: Supplementary file 1 — Supplementary figures. [file thnov10p8430s1.pdf]

Supplementary Information

Figure S1.

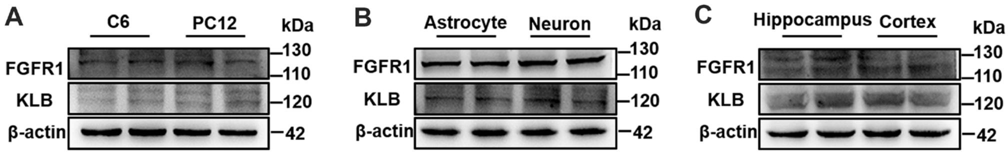

Figure S1. The expression of FGFR1 and KLB in brain tissues and cells. The expression levels of FGFR1 and KLB in C6 astrocytes and PC12 neurons (**A**), primary astrocytes and primary neurons (**B**), and brain tissues (**C**) were detected by western blot.

Figure S2.

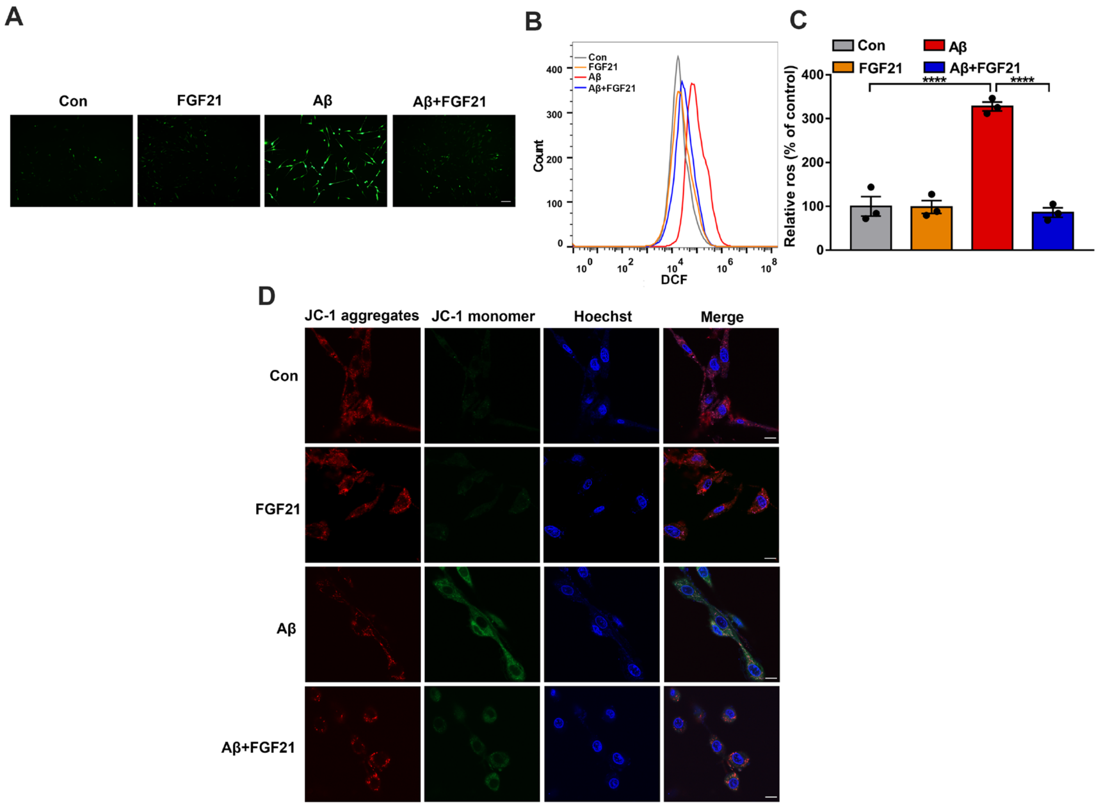

Figure S2. Effect of FGF21 on Aβ(25-35)-induced mitochondrial dysfunction in PC12 cells in a co-culture *in vitro* model. A-B. PC12 cells in a co-culture *in vitro* model were stained with DCFH-DA probe for detection of intracellular ROS levels, and fluorescence microscopy (**A**) and flow cytometry (**B**) were used for analyses. Representative images are shown. Scale bar, 50 μm. **C.** Quantitative results for **B**. n=3. **D.** PC12 cells in the *in vitro* co-culture model were stained with the JC-1 probe for detection of the mitochondrial membrane potential, and confocal laser scanning microscopy was used for analysis. Scale bar, 10 μm. Data are presented as the mean ± SEM. **** $p < 0.0001$.

Figure S3.

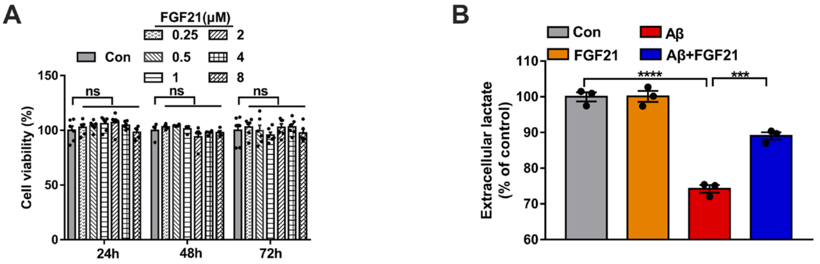

Figure S3. Effects of FGF21 treatment alone on C6 cells, and analysis for lactate levels in medium from C6 cells treated with Aβ(25-35) and/or FGF21. A. FGF21 (0.25 μM, 0.5 μM, 1 μM, 2 μM, 4 μM, 8 μM) was added to C6 cells, and 24/48/72 h later, the cell viability of the C6 cells was detected by MTT assays. *n*=6. ns, not significant. **B.** Cells were treated with Aβ(25-35) and/or FGF21, and after 48 h extracellular lactate levels in medium from C6 cells were detected. *n*=3. All data are presented as mean ± SEM. *** $p < 0.001$; **** $p < 0.0001$.

Figure S4.

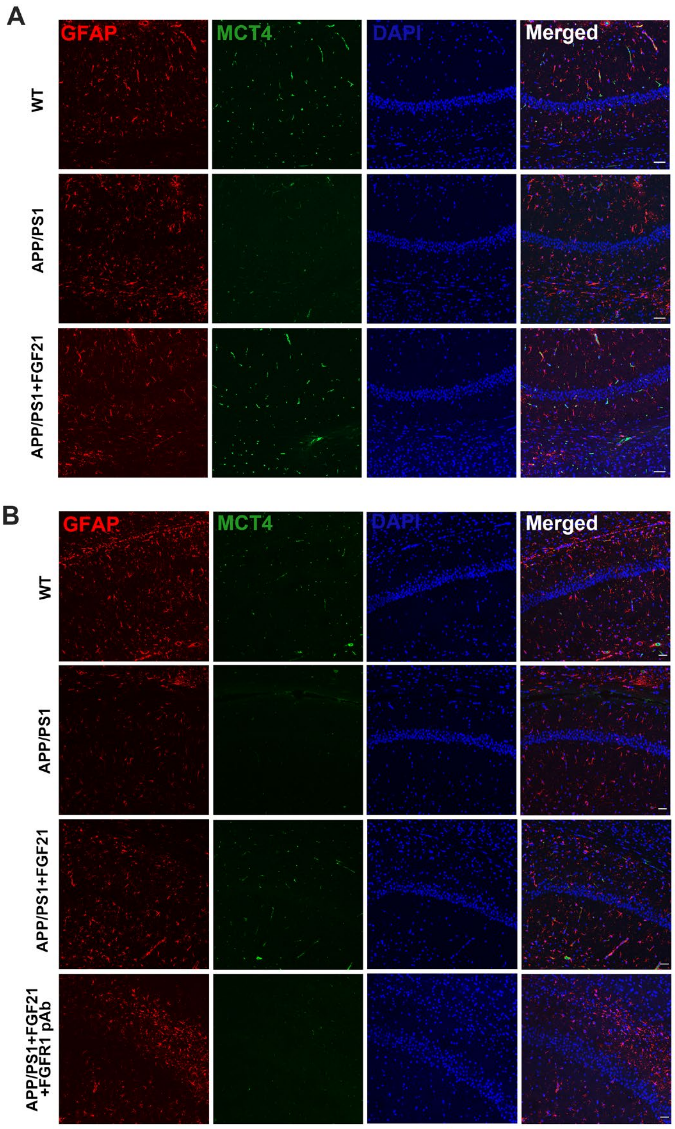

Figure S4. Immunofluorescence staining of MCT4 in the mouse brain. A. In peripheral administration experiments using transgenic mice, mouse brain slices were costained with anti-MCT4 antibody, anti-GFAP antibody, and DAPI. Scale bar, 50 μm. **B.** In central administration experiments using transgenic mice, mouse brain slices were costained with anti-MCT4 antibody, anti-GFAP antibody, and DAPI. Scale bar, 50 μm.

Figure S5.

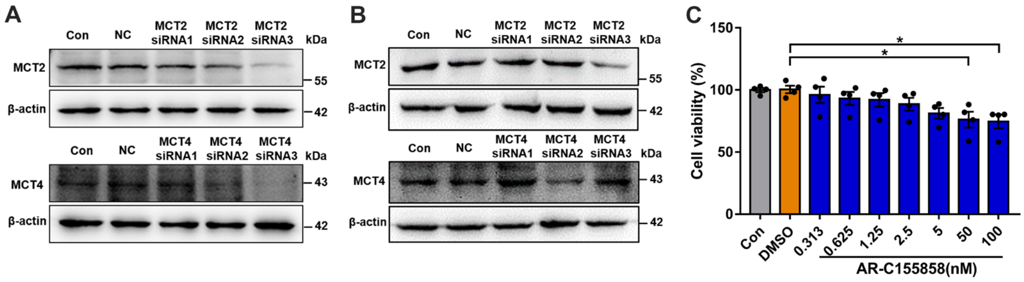

Figure S5. Silencing efficiency test of MCT siRNA and cytotoxicity assay of MCT2 inhibitor. A. In *in vitro* transfection experiments, silencing efficiencies of synthetic siRNAs for MCT2 and MCT4 were tested by western blot. **B.** In *in vivo* transfection experiments, silencing efficiencies of synthetic siRNAs for MCT2 and MCT4 were tested by western blot. **C.** Effects of different concentrations of MCT2 inhibitor (AR-C155858) on cell viability were assessed by MTT assays. n=4. Data are presented as the mean ± SEM. * $p < 0.05$.

Figure S6.

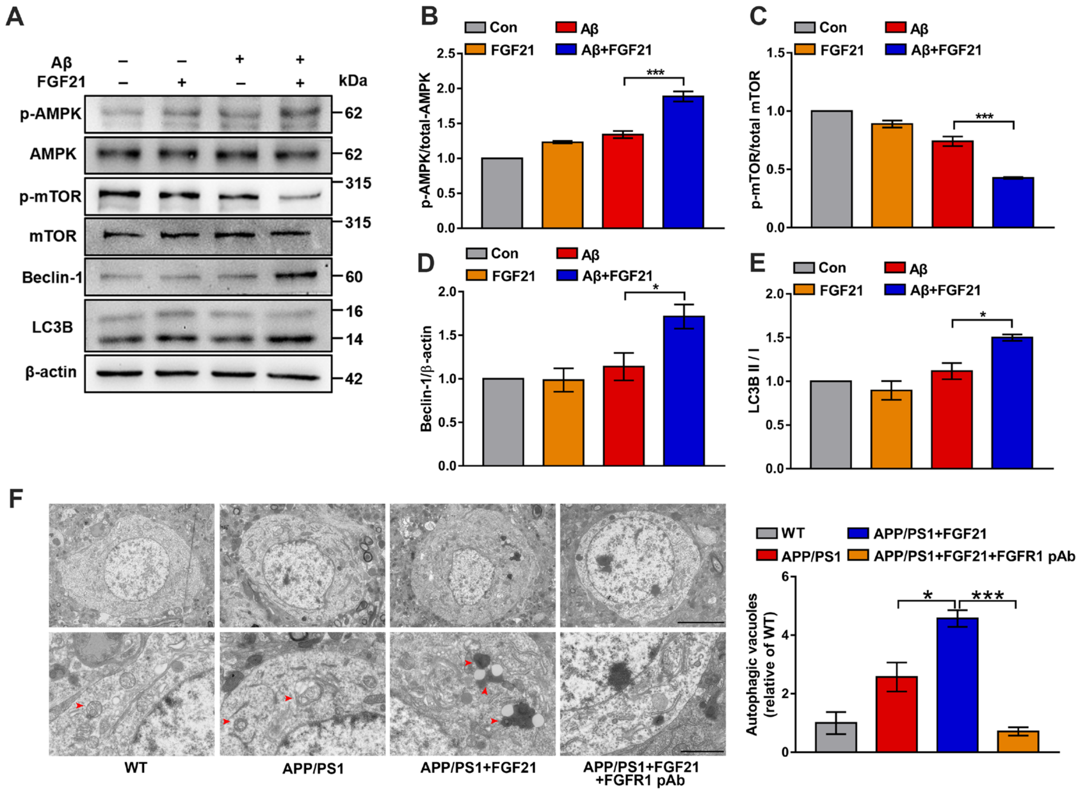

Figure S6. Effect of FGF21 on autophagy in PC12 cells in an *in vitro* co-culture model induced by Aβ(25-35). A. The expression levels of p-AMPK, AMPK, p-mTOR, mTOR, Beclin-1, and LC3B in PC12 cells co-cultured in an *in vitro* model were detected by western blot. Representative images are shown. **B-E.** Quantitative results for **A**. *n*=3. **F.** In APP/PS1 mice with/without ICV administration of FGF21 or FGF21+FGF21 pAb, hippocampal autophagic vacuoles were analyzed using electron microscopy. Scale bar, upper images: 5 μm, lower images: 2 μm. All data are presented as the mean ± SEM. * $p < 0.05$; *** $p < 0.001$.
